# Investigating the synergistic effects of Metakaolin and silica fume on the strength and durability of recycled aggregate concrete at elevated temperatures

**DOI:** 10.1038/s41598-025-11494-w

**Published:** 2025-08-12

**Authors:** Imran Haider, Muhammad Yaqub, Inamullah Inam, Tariq Ali, Muhammad Zeeshan Qureshi, Nabil Ben Kahla, Anwar Ahmed, Hawreen Ahmed

**Affiliations:** 1 Department of Civil Engineering, University of Engineering and Technology, Taxila, Pakistan; 2 Department of Civil Engineering, Engineering Faculty, Laghman University, Mehtarlam, Afghanistan; 3 Department of Civil Engineering, Swedish College of Engineering and Technology, Wah, 47080 Pakistan; 4 Civil Engineering Department, College of Engineering, King Khalid University, Abha, Saudi Arabia; 5 Center for Engineering and Technology Innovations, King Khalid University, Abha, 61421 Saudi Arabia; 6 Department of Civil Engineering, College of Engineering, Northern Border university, Arar, Arar, 73222 Saudi Arabia; 7 Department of Highway and Bridge Engineering, Technical Engineering College, Erbil Polytechnic University, Erbil, 44001 Iraq

**Keywords:** Silica fume, Metakaolin, Fire resistance, Mechanical properties, Durability properties, Environmental sciences, Engineering, Materials science

## Abstract

The use of recycled aggregate (RA) as a partial or full replacement of natural aggregate (NA) is a suitable method of concrete production that has positive impacts on the environment. However, recycled aggregate concrete (RAC) has relatively lower strength and durability than that of normal concrete. To improve concrete performance, silica-fume (SF) was added with 2.5% increment up to 7.5% and metakaolin (MK) is added with a 2.5% decrement from 15 to 7.5%. The concrete with 50% RA, 10% MK and 5% SF showed notable advancement in performance after 28 days of curing. At 28 days of curing, the concrete samples had 31.5 MPa compressive strength, 5.7 MPa splitting tensile strength, and 10.6 MPa flexural strength, a strength improvement of 5.19%, 16.47%, and 8.52% over control concrete. Ultrasonic pulse velocity (UPV) indicated a 16.13% increase alongside a 20.87% reduction in water absorption which confirmed stronger bond performance and better durability of modified concrete. RCA content influences acid resistance negatively when reaching 75% RCA shows maximum deterioration. In addition, the fire resistance of such concrete resulted in higher performance at different temperature conditions for the concrete. This is due to the small particles of silica fume and metakaolin which acted as major factors and led to performance enhancements by filling in the concrete matrix gaps. The combination provides affordable, sustainable construction alternatives. Experiments show that SCMs can produce high-performance recycled concrete for modern building construction.

## Introduction

In recent years, the production of cement has boosted due to the urban development and infrastructure projects^1,2^. As a result of this raised demand, global cement production needs to respond greatly to production requirements^3^. Cement production runs into billions of tons, for instance in 2018 reaching 4,111.1 million tons^4^ (second most used commodity after water^5^). Whilst it is an energy intensive process, the manufacture of cement causes about 5% of carbon dioxide (CO2) production around the globe^6^. As the demand continues to increase, cement consumption is expected to increase by 8%^7^, leading to an increasing amount of embodied emissions in the construction sector. Therefore, to tackle this, industry and research experts have drawn attention towards the fact that by incorporation supplementary cementitious materials (SCMs) in the cement as the substitution up to 25% can be an efficient way for decreasing the carbon environmental impact of the construction activities^8,9^. Similarly, massive quantities of concrete waste are also being produced in new urbanization efforts as well as in restoration projects on a global scale. The demolition of concrete rubble is also dealt with as an environmental threat^10^. However, the waste material can be recycled for use as valuable construction material, including for the production of concrete, as an alternative source of aggregates^11^. Processing of construction and demolition debris to produce recycled aggregates involves extraction of dumped concrete, crushing, grading and cleaning of the resulting aggregates to mitigate contaminants and fine particles^12,13^. Using recycled aggregates in concrete and partially replacing the cement with SCMs helps to reduce the overall environmental loading of construction projects^14,15^. In addition, current research indicates that the use of carbon dioxide (CO2) curing significantly increases the environmental sustainability of the recycled concrete aggregate (RCA)^16,17^. Usually, recycled aggregates mainly contain a 65-80% of virgin coarse aggregates and 35%-20% covered with aged mortar paste^18^. Recycled concrete aggregates performance is highly sensitive to characteristics of original parent concrete, such as proportioning and workability^19^. Concrete produced with RCA has lower mechanical properties and less durability compared to that of concrete which is made using virgin aggregates, such as limestone or granite^20^. It is suggested that loss in performance is probably related to the more porosity of RA and the weak ITZ produced between the fresh and aged concrete components^21,22^. A second issue regards the durability of RA in concrete over long time periods, with aggregates degrading in different environmental conditions^23^.

To overcome these shortcomings incorporating mineralogical admixtures and pozzolanic materials as partial replacement for cement in the formulation of RAC can make a significant contribution to improvement in properties of recycled concrete aggregates^24–26^. These additives are utilized to plasticize the open pore network of processed aggregates with calcium-silica-hydrate (C-S-H) gel to densify the recycled aggregates concrete (RAC) matrix and advance both its mechanical behavior and durability^27,28^. Silica fume and metakaolin are effective pozzolans that have been shown to improve concrete properties, i.e. enhance their strength and durability^29^. Despite that, supply of SCMs, particularly in some remote geographical areas, may not be available to indeed upcycle concrete^30^. The surplus demand for SCMs induced by strict environmental regulations increases the severity of shortage in SCMs^31^. For example, the policies promoting alternative energy sources in the state of Miami, USA, have caused a large reduction of the supply of a common SCM fly ash^32^. Therefore, the potential adverse effects of concrete production can be significantly reduced by the increased use of SCMs, reusing recycled concrete aggregates and by employing state-of-the-art curing methods during concrete production. Not only do these strategies diminish our need for natural resources but they also cut back on carbon footprint and environmental risk. The potential for sustainable and durable construction material is demonstrated through the application of mineral admixtures and pozzolanic materials in recycled concrete aggregates. Experiments have shown that introducing mineral admixtures, SCMs, or fibers to RAC can provide distinct benefits. Also, the probability for the structure to experience the fire is always present due to many reasons. Therefore, it is also necessary to check the fire performance of such modified concrete to access the behavior i.e. strength reduction rate or residual strength. There has been no major study on fractional replacement of cement by using silica-fume and metakaolin in a combination and utilizing them in recycled aggregate concrete to study mechanical behavior, durability and fire performance. The durability or resilience parameter of concrete indicate the originality for current study. To assess the mechanical properties compressive strength, tensile strength and flexural test was performed. Durability was assessed by UPV, water absorption and acid resistance. Finally, to crosscheck the lab results, statistical analysis was performed. This study will assist readers to build knowledge on increasing the qualities of RAC by utilizing waste materials (SF-MK and recycled aggregates) to enhance the characteristics and sustainability of such concrete.

## Materials and methods

### Binders

**Fig. 1:**
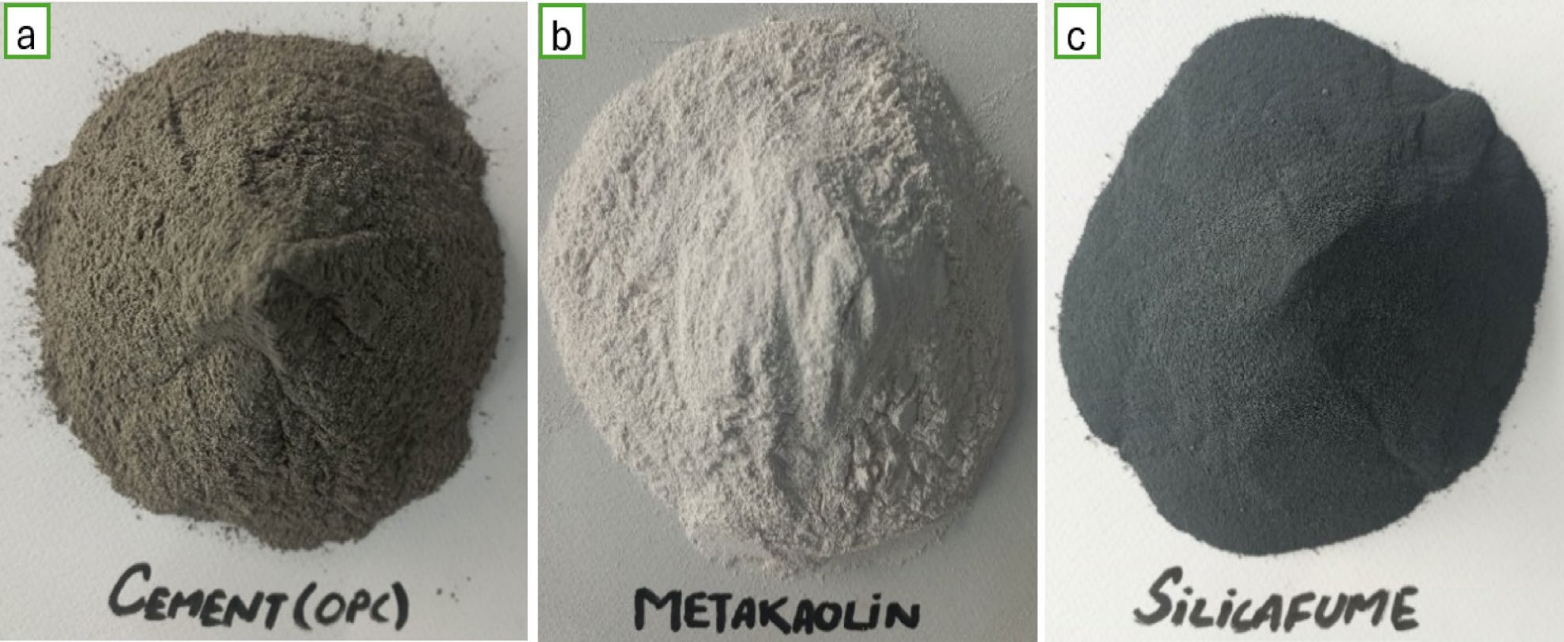
(**a**) Cement (OPC); (**b**) Metakaolin; (**c**) Silica-fume.

Type I cement was employed in this study in accordance with ASTM C150^33^. Recently, as a mineral admixture there has been a spike in interest in using MK for similar purposes^34^. B. Sabir^35^ has given detailed insight of the research on the use of MK acting as partial substitute of cement in mortar as well as in concrete. Metakaolin (MK) can enhance the performance of cementing composites by increasing their pozzolanic reactivity, similar to silica fume (SF). SF concrete shows higher durability and mechanical characteristics, whereas MK concrete is more affordable yet easier to deal with. Metakaolin is a substance based on thermally activated alumino-silicate prepared by calcining kaolin clay at temperatures between 650-800^o^C^35^. It generally contain 40–45% Al_2_O_3_, 50–55% of SiO_2_ and is highly reactive. MK is far more different from natural pozzolans and other manufactured pozzolans available in other forms in which it is a main product, whereas Silica fume and Fly Ash are secondary. MK can be manufactured under regulated conditions to acquire specific qualities. Experimentally it is verified that concrete containing 10% of Metakaolin had a greater compressive strength than control concrete at all ages up to 180 days^36^. As compared to Silica-fume concrete for the same amount of replacement, MK concrete demonstrated rapid strength growth at 7 and 14 days but had comparable strength after 28 days^36^. In terms of endurance, MK and SF concrete was shown to be much more resistant to chloride penetration than the normal concrete^36^. The binder (OPC, MK and SF) used in this study are shown in Fig. 1. After reviewing available research^35^, it can be said that MK is a very useful and effective pozzolan. It is less expensive yet has the same pozzolanic reactivity as silica fume. MK particles typically measure less than 3 μm which is smaller than OPC particles but larger when compared to SF. When incorporated as a fractional substitution of cement in concrete, it enhances early strength without compromising long-term strength. MK enhances concrete performance by reacting with Ca (OH)_2_, forming additional C-S-H. MK strong reactivity and white color make it ideal for appearance-matching and architectural applications, since it does not discolor concrete like SF, which has limited tonnage.

Additionally, it improves impermeability and hazardous ion diffusion. Several research projects have been undertaken to alter the microstructure and study the hydration process of cement pastes containing MK^37,38^. These investigations reveal that at early stages of hydration process, the pozzolanic reaction rate is faster in MK pastes when compared to SF pastes but retracts in MK pastes during protracted curing. Using MK in cement matrix with a greater water-to-binder ratio (e.g., w/b = 0.55) leads to lower pore diameters but increased overall porosity^39^. When employed in low water-to-binder ratio i.e. high-performance cement pastes, it decreases pore size and hence overall porosity^38^. Properties of OPC, MK and SF can be seen in Table 1 below:

**Table 1:** Physical and chemical properties of OPC, Metakaolin and Silica-fume.

	Particles Size (µm)		Setting Time (min)				Soundness (%)		Consistency (%)		Fineness (m^2^/kg)	
			Initial		Final							
Physical Propert ies	OPC	≤ 75	36		415		1.8		34.0		381	
	Metakaolin	≤ 75	-		-		-		-		12,482	
	Silica-fume	≤ 75	-		-		-		-		15,765	
Chemical Properties		LOI	Al_2_O_3_	Fe_2_O_3_	SiO_2_	Na_2_O	CaO	SO_3_	P_2_O_5_	K_2_O	MgO	TiO_2_
	OPC	2.60	4.1	2.1	22.3	1.4	62.4	2.3	-	1.8	3.6	-
	Metakaolin	1.43	42.25	1.42	51.34	0.1	0.13	-	0.06	0.75	0.37	2.15
	Silica-fume	3.06	0.93	1.15	91.26	-	1.10	0.85	-	-	1.65	-

### Fine and coarse aggregate

**Fig. 2:**
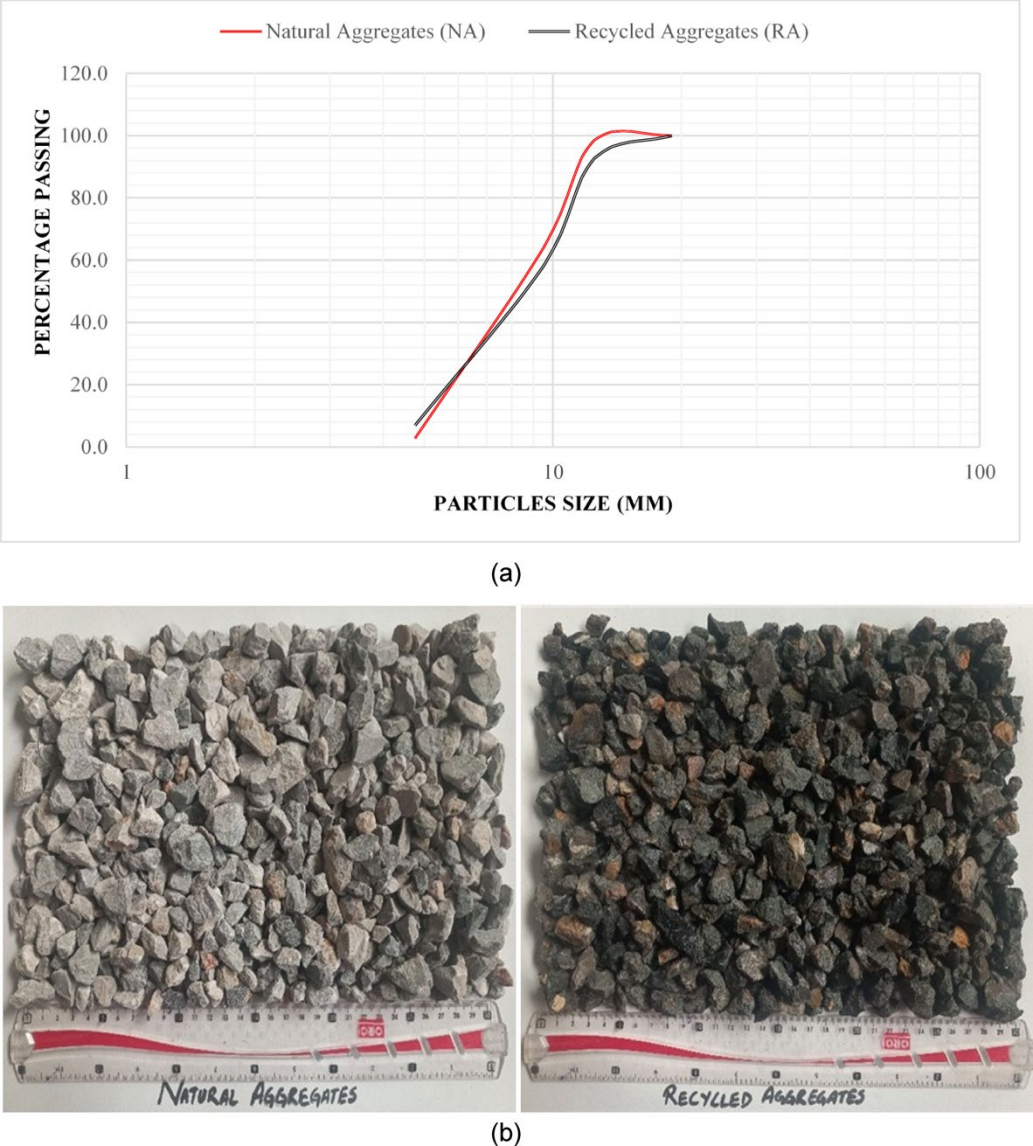
(**a**) Particles size distribution of Natural Aggregates and Recycled Aggregates (**b**) Natural Aggregates vs. Recycled Aggregates.

Quarried sand was utilized as a fine aggregate having a fineness modulus of 2.73, while crushed stone was used as a coarse aggregate. During and after World War-II, incorporation of C&D Waste as aggregates in new concrete was adopted due to the large amount of ruins and debris created by bombardment in urban areas, especially in the UK and Germany^40^. RAC then showed to have more water absorption, lower compressive strength, less freeze and thaw resistance, and less dry shrinkage than NAC^39^;^41^. There are a growing number of studies on RA and RAC as people become more conscious of environmental issues and the need for sustainable development. Natural aggregates was acquired from the local crusher plant and before being included into the concrete they were wet while Fine aggregates and recycled coarse aggregates were employed on saturated surfaces condition. Table 2 provides information on natural and recycled aggregates, whereas Fig. 2 (a) show grading curves for NA and RCA and Fig. 2 (b) shows natural vs. recycled aggregates, respectively. ASTM C 128^42^ and C 127^43^ were used to determine the specific gravity of both coarse and fine aggregates.

**Table 2:** Physical properties of fine and coarse aggregates.

	Fine Aggregates	Coarse Aggregates	Recycled Aggregates
Abrasion Resistance (%)	-	17.42	31.78
Fineness Modulus	2.59	3.37	3.94
Water Absorption (%)	4.33	3.19	5.10
Specific Gravity	2.55	2.73	2.27
Bulk Density (kg/m^3)^	1524	1620	1491

### Admixture

**Fig. 3:**
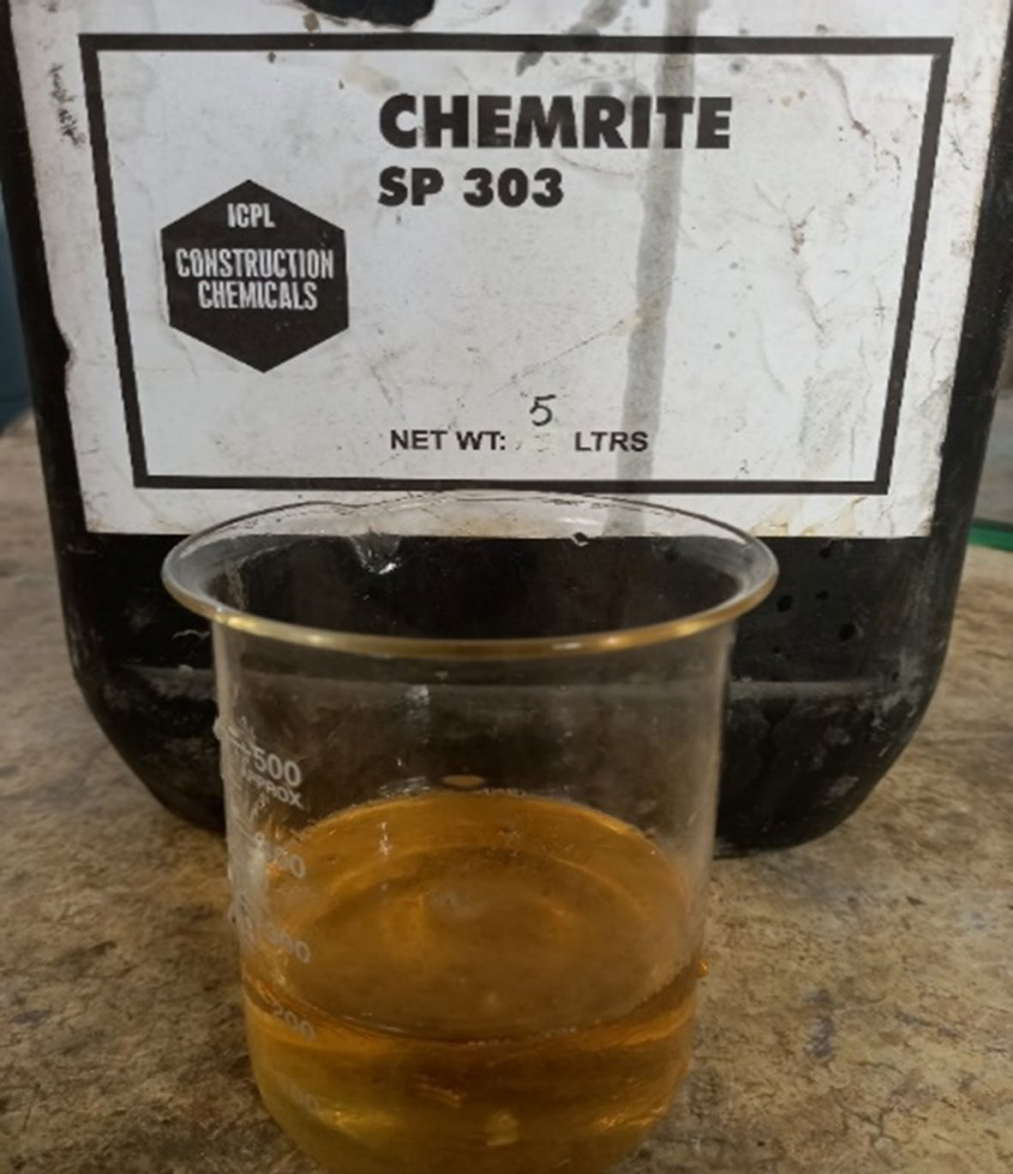
Super-Plasticizer.

As a high-range water-reducing (HRWR) additive, a polycarboxylate-based third-generation superplasticizer is used in concrete (Fig. 3), by using the reference of ASTM C494^44^, to enhance workability or flowability. The additive was mixed to all concrete mixes at a uniform rate of 0.75% (by binder weight).

## Mixed proportions

A total of twenty mixes were prepared by using recycled aggregate (RA) at varying levels (0, 25, 50, and 75%) instead of virgin coarse aggregates. Metakaolin was introduced in decreasing order with an interval of 2.5% from 15 to 7.5% and silica-fume was added in increasing order with an interval of 2.5% from 0 to 7.5% as a cement alternative and mix design is further elaborated in Table 3. Preliminary laboratory testing and existing literature indicate that the ideal cement replacement amount is around 10–15%^35,45^ of both admixtures.

**Table 3:** Mix design of concrete samples.

Mix ID	Cement (kg/m^3^)	Metakaolin (kg/m^3^)	Silica-fume (kg/m^3^)	Sand (kg/m^3^)	Coarse Aggregates (kg/m^3^)		Water (liter/m^3^)	Admixture (liter/m^3^)
					NA	RA		
R0-M0-SF0	350	-	-	750	1100	-	175	1.31
R0-M15-SF0	297.5	52.5	-	750	1100	-	175	1.31
R0-M12.5-SF2.5	297.5	42.7	8.75	750	1100	-	175	1.31
R0-M10-SF5	297.5	35	17.5	750	1100	-	175	1.31
R0-M7.5-SF7.5	297.5	26.25	26.25	750	1100	-	175	1.31
R25-M0-SF0	350	-	-	750	825	275	175	1.31
R25-M15-SF0	297.5	52.5	-	750	825	275	175	1.31
R25-M12.5-SF2.5	297.5	42.7	8.75	750	825	275	175	1.31
R25-M10-SF5	297.5	35	17.5	750	825	275	175	1.31
R25-M7.5-SF7.5	297.5	26.25	26.25	750	825	275	175	1.31
R50-M0-SF0	350	-	-	750	550	550	175	1.31
R50-M15-SF0	297.5	52.5	-	750	550	550	175	1.31
R50-M12.5-SF2.5	297.5	42.7	8.75	750	550	550	175	1.31
R50-M10-SF5	297.5	35	17.5	750	550	550	175	1.31
R50-M7.5-SF7.5	297.5	26.25	26.25	750	550	550	175	1.31
R75-M0-SF0	350	-	-	750	275	825	175	1.31
R75-M15-SF0	297.5	52.5	-	750	275	825	175	1.31
R75-M12.5-SF2.5	297.5	42.7	8.75	750	275	825	175	1.31
R75-M10-SF5	297.5	35	17.5	750	275	825	175	1.31
R75-M7.5-SF7.5	297.5	26.25	26.25	750	275	825	175	1.31

## Mixing procedure

**Fig. 4:**
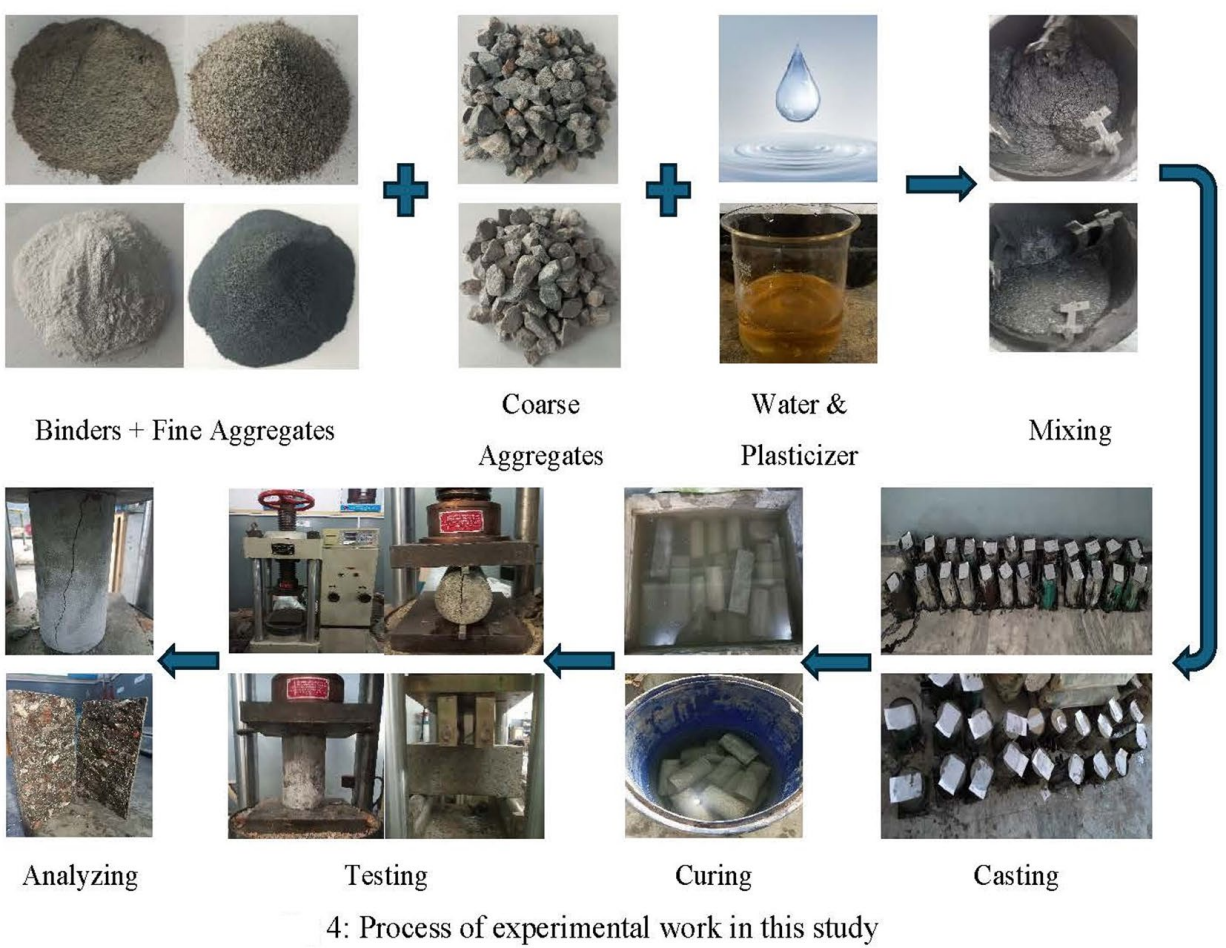
Process of experimental work in this study.

A mechanical mixer was used to mix metakaolin, silica-fume, cement, fine aggregate and coarse aggregate (NA and RA) in dry form for 120 s having a mean speed of 70 rpm. Then, at 70% completion of the mixing process, water was induced and mixed again for seventy seconds. The leftover water and additives was poured into the blend afterward then blend at 100 rpm for an additional 60 s. After 30 s of repose, the concrete blend was re-blended for another 60 s before casting. First the concrete samples were cast as per scheme then removed from the molds after 24 h and put in a water tank maintained at a temperature of 22^o^C until fully cured referring to ASTM C511-21 [46] . To restrict the minerals leaching from concrete samples, water was treated with calcium hydroxide (hydrated lime). A minimum of three samples were made for every concrete mix to take average experimental results as the final value. A complete experimental process is shown in Fig. 4 below:

## Flow chart of research methodology


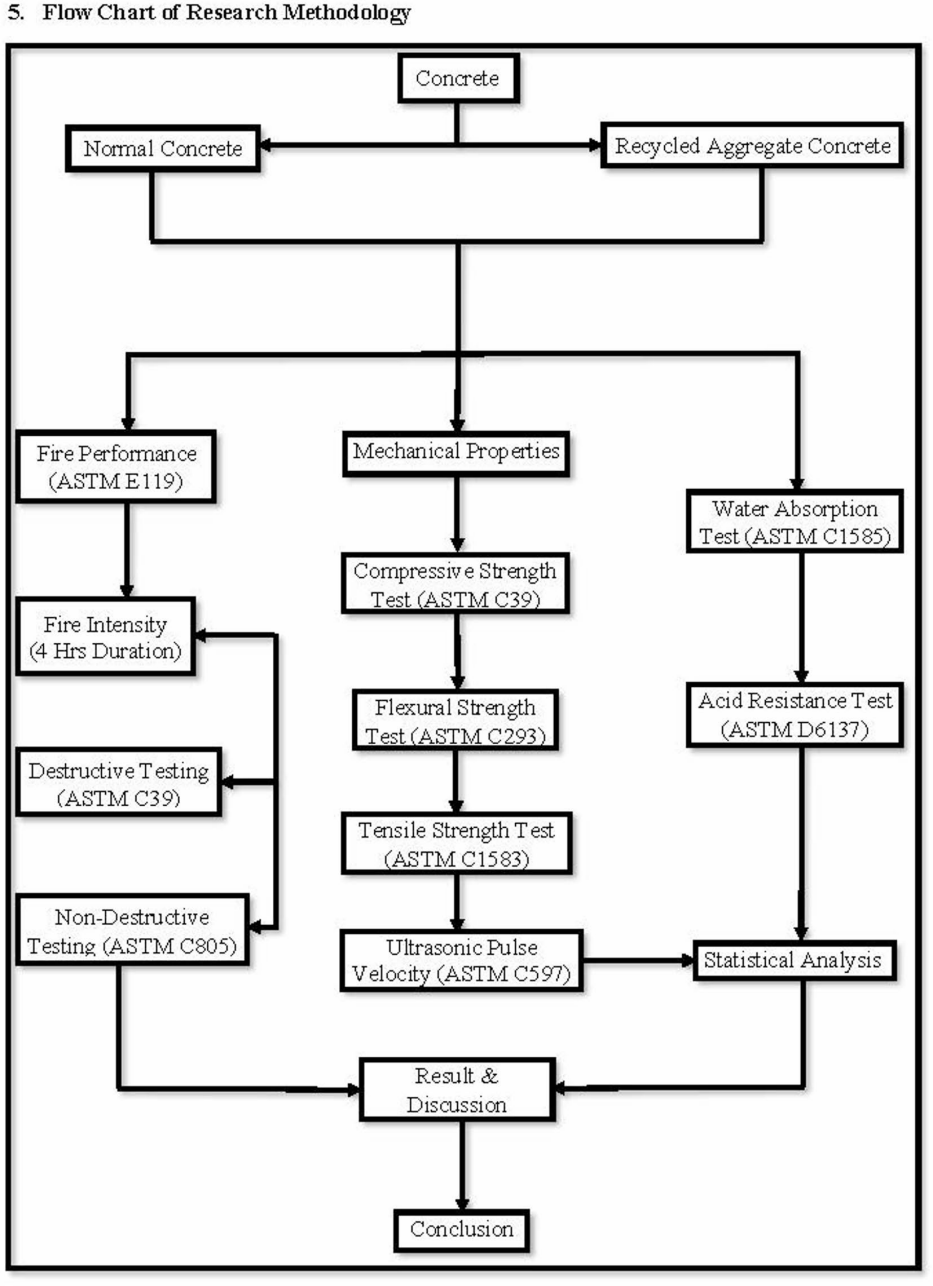


## Result and discussion

### Compressive strength

**Fig. 5:**
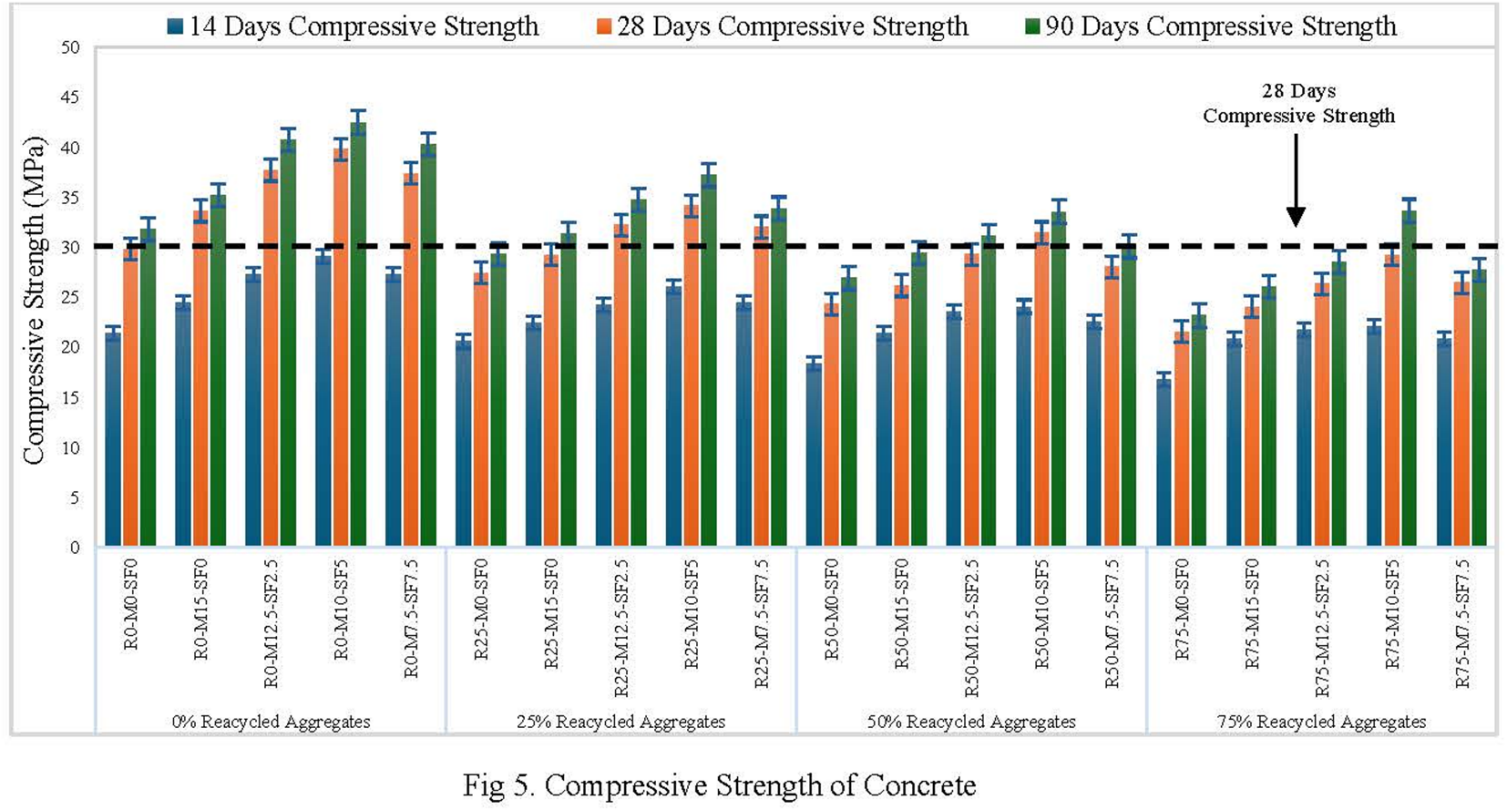
Compressive Strength of Concrete.

Figure 5 demonstrates the impact of RA, metakaolin, and silica-fume on the compressive strength of concrete at different curing ages using compression machine (Fig. 7a). RA was utilized as a replacement for NA at four levels: 0%, 25%, 50% and 75%. At every replacement percentage and curing stage, blends with both MK and SF had the highest strength, followed by blends with 10% MK and 5% SF. The reference specimens without MK and SF addition had the lowest compressive strength values. Eventually the compressive strength of concrete samples increased with passage of time in all formulations. For 50% RA and 28 days of curing, the compressive strength increased by 23.52% with 10% MK and 5%SF replacement in contrast to the strength of 14 days curing. After 90 days of curing, concrete compositions containing 50% recycled aggregates (RA) showed a 28.3% increase in strength, with MK and SF substituting 10% and 5% of the cement, respectively. Concrete’s compressive strength increased with 10% MK and 5% SF addition to concrete as compared to control samples i.e. 0% addition of MK-SF at 14, 28, and 90 days of curing. It is formulated that MK and SF, a byproduct of cement hydration, react with Ca (OH)_2_ to form additional C-S-H gel, which increases concrete strength and density.

This response lowers C-H concentration, boosting long-term endurance. Their small particles fill micro-voids between cement grains, reducing porosity and enhancing the interfacial transition zone. It also adds more nucleation sites for hydration product precipitation, accelerating C-S-H crystallizations and resulting in a denser microstructure. These components help to increase rapid densification and strength during the curing process. Blends with MK and SF have somewhat higher strength than blends with recycled aggregates only, which helps to counteract the detrimental effects of RA on concrete strength. The increase is significant for mixes that contain 10% MK and 5% SF. This is because MK and SF improve microstructure, accelerate the hydration process, and raise concrete density, resulting in increased load capacity. The addition of recycled aggregate affected the integrity of concrete, although increasing the MK and SF dose not only balance the impact but also slightly boosted the strength when compared to the control sample. The surface area might impact MK and SF as it increase the density of the matrix, reduces the pores, and reduce the permeability, as a result compressive strength is improved^45^.

**Fig. 6:**
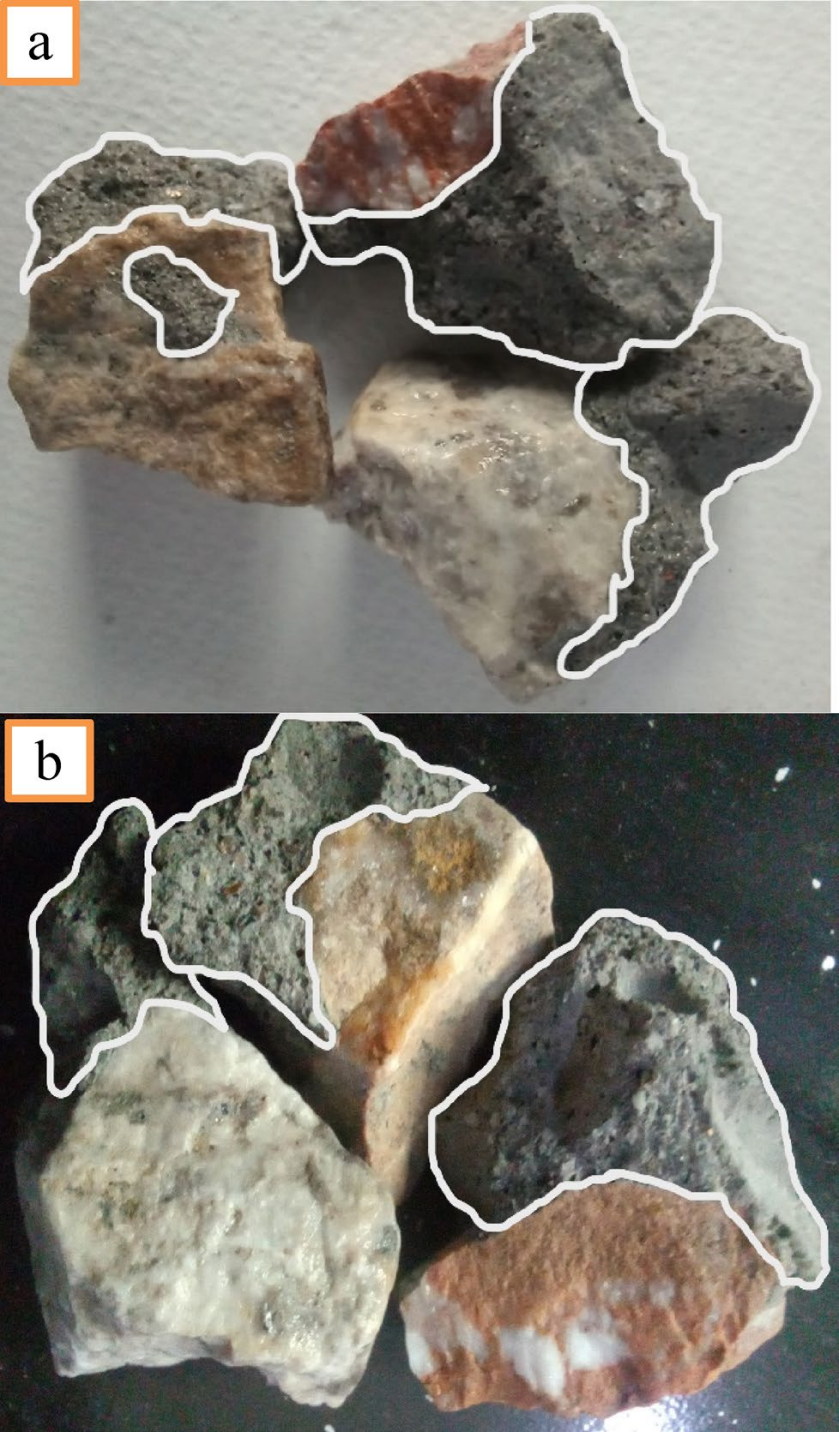
(**a** & **b**) Old attached mortar with coarse aggregates.

Concrete compressive strength decreases as more normal-weight material is replaced with recycled aggregate (RA). When recycled concrete aggregate (RAC) is replaced up to 75%, 21.8-27.1% of strength reduction is seen when compared to concrete with control concrete samples (R0-MK0-SF0) across all evaluated specimens. At 50% replacement of NA with RA, a 14.2-18.4% drop in strength is seen for (MK0-SF0). The reduction in strength is due to aged mortar surrounding the surface of RA as shown in Fig 6: (a & b). Three types of interfacial zones (ITZ) are mainly present in recycled aggregate concrete^47^.

The first ITZ is present between the new binder and recycled aggregate^48^, The second one lies between old and fresh binder^32^, and the third one lies in between RA and attached aged binder^49^. Low mechanical strength is subjected to a hair-like crack in the third interfacial zone when compared to the samples produced with virgin aggregate^50^. On whole, the outcomes suggest that MK and SF stimulates the production of additional phases, which strengthens the microstructure, thus fully overcome the weaker interfacial transition zones formed. Hence, the combination of MK and SF permits the substitution of natural aggregates up to 50% with RA and a slight increase in strength parameters gaining after 90 days of production (RA-0 no MK-SF 31.8 MPa; RA-50, with MK10-SF5 33.6 MPa).

**Fig. 7:**
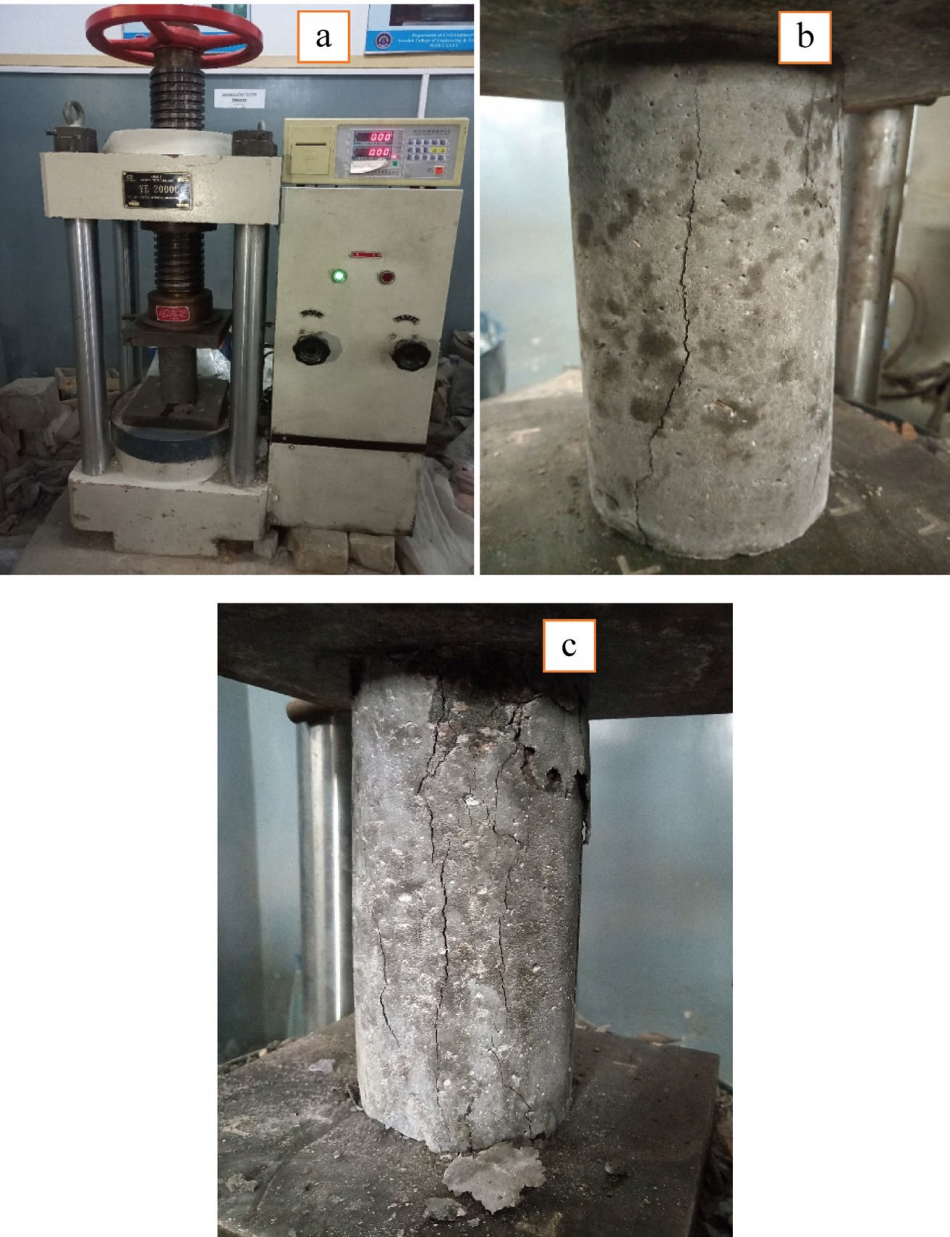
(**a**) Specimen under testing machine; (**b**) Crack in Natural Aggregate Concrete; (**c**) Crack in Recycled Aggregate Concrete.

#### Statistical analysis of compressive strength

**Fig. 8:**
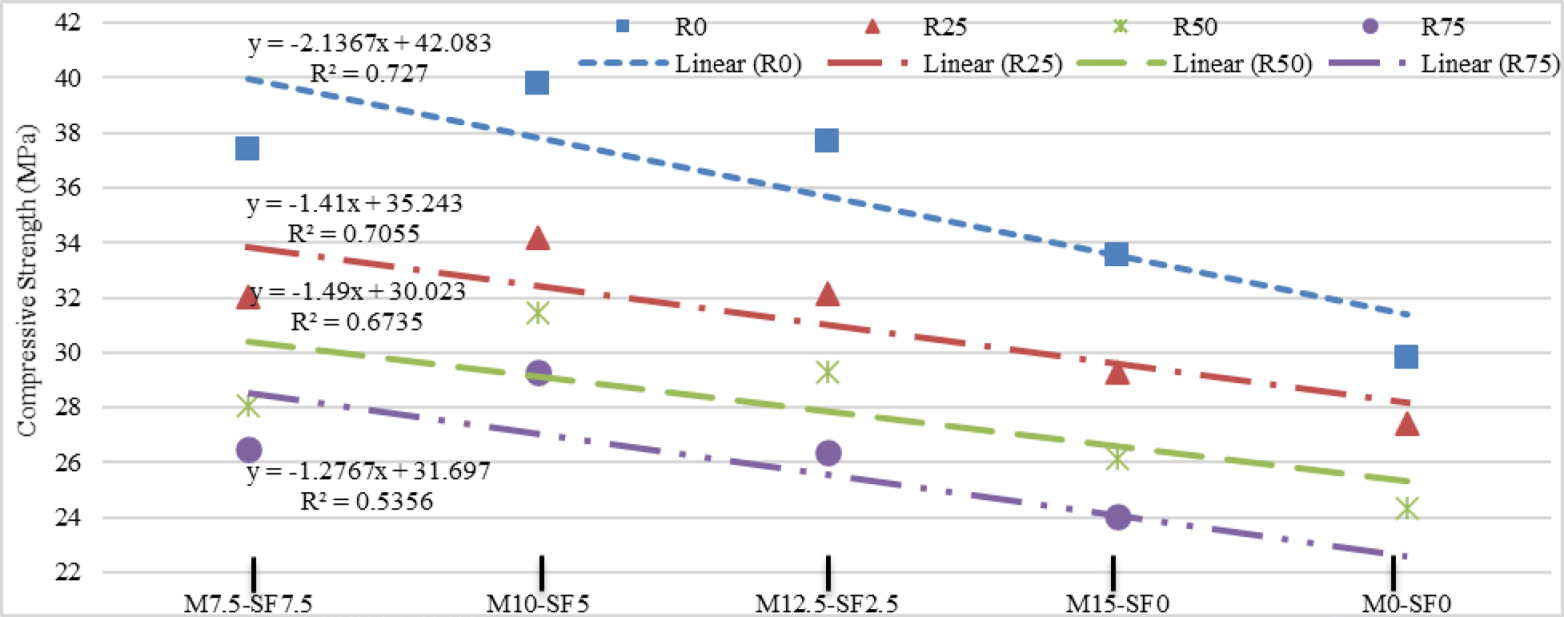
Statistical Analysis of Compressive Strength of Concrete.

Figure 8 depicts the compressive strength at the curing of 28 days for each blend. Every point on the graph reflects the mean value of three samples results. A basic statistical analysis for the obtained results was also carried out, and to the plot equation a linear fit curve was produced for all data series. Figure 8 reveal that the highest compressive strength was achieved with no incorporation of recycled aggregate, as compared to seventy-five (75%) percent of substitution, regardless of MK-SF addition. Exactly due to the recycled aggregate’s porous nature, which resulted in a higher proportion of water absorption. Figure 8 of statistical analysis shows the actual links between the tested variable and the R-squared result. When the R-squared value approaches one (1), it indicates the best regression response. In majority of statistical analyses, the R-square value is greater than eighty (70%) percent, indicating the good match between metakaolin, silica-fume, and recycled aggregates, hence shows that MK and SF are performing their function to overcome the inferior impact of recycled aggregate. However, in a few scenarios, the value of R-square was less, which is the result of using too much addition of SCMs and RA causing deep impact on the hydration processes and concrete matrix formation. Regardless of the combinations, combining metakaolin and silica fume at the same time enhances compressive strength marginally. This might be because MK-SF enhances the microstructure^51^. Examining the failure patterns of the control and modified concrete samples during the compression strength test, the fractures in the modified samples passes at various locations along their whole length (Fig. 7c). In contrast, control samples failed around the middle across the length (as shown in Fig. 7b).

### Split tensile and flexural strength

**Fig. 9:**
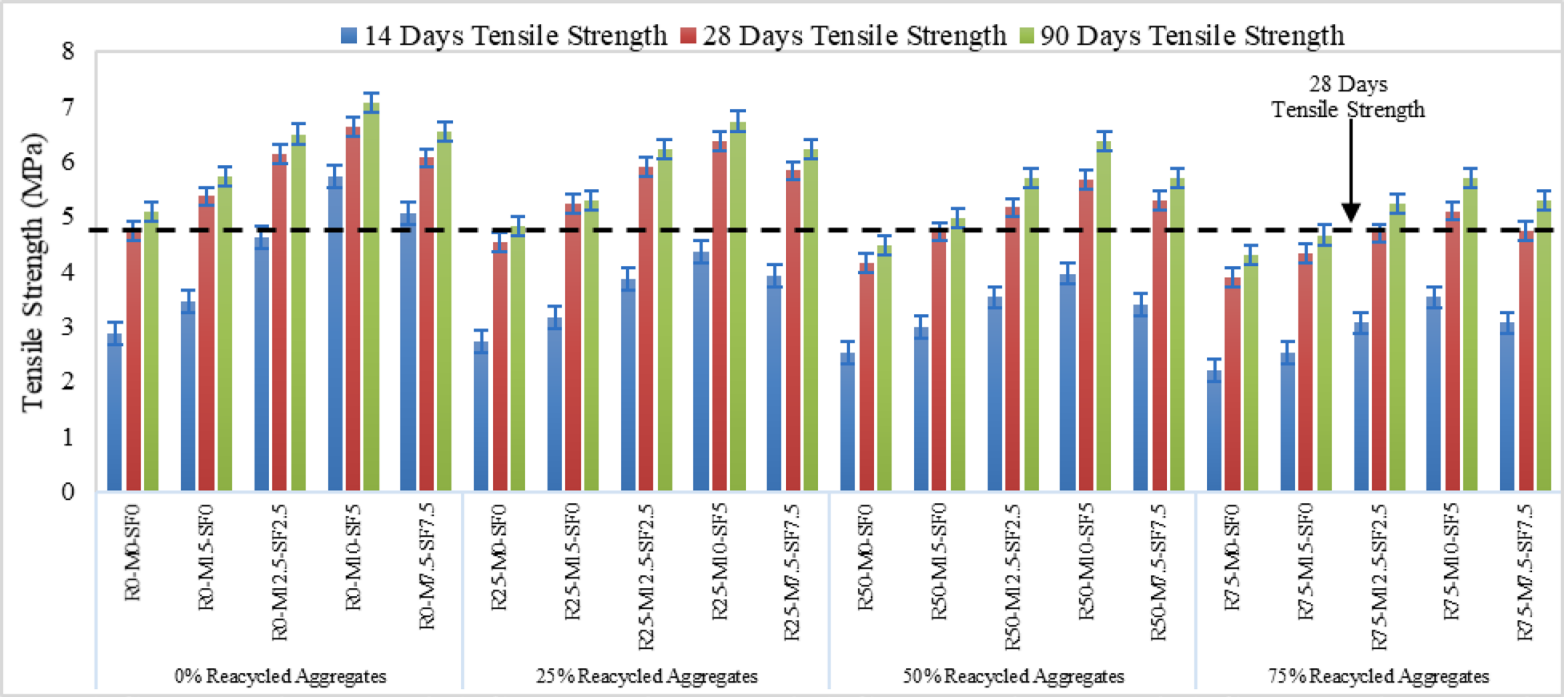
Tensile Strength of Concrete.

**Fig. 10:**
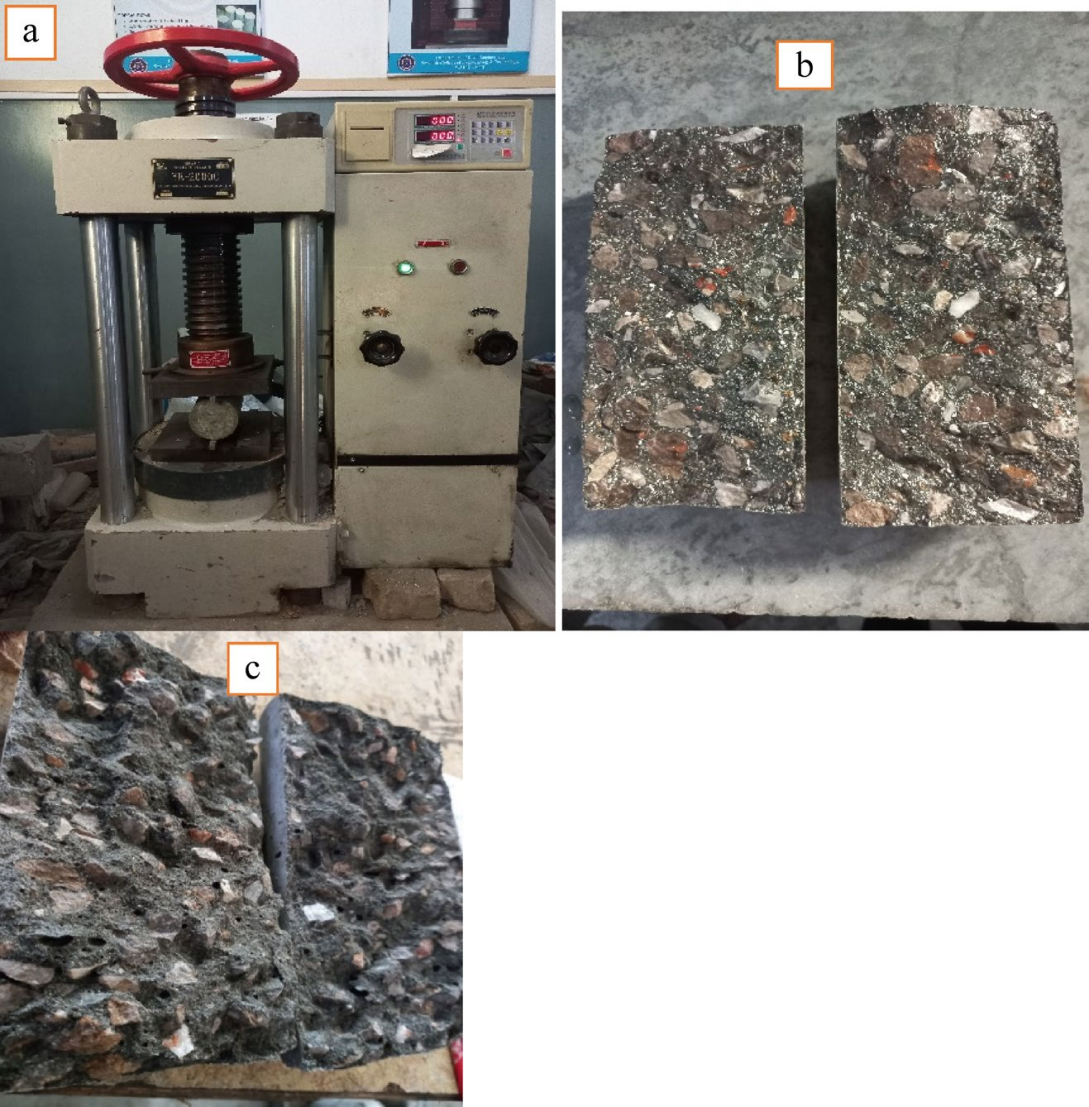
(**a**) Specimen under testing machine; (**b**) Crack in Natural Aggregates Concrete; (**c**) Crack in Recycled Aggregates Concrete.

**Fig. 11:**
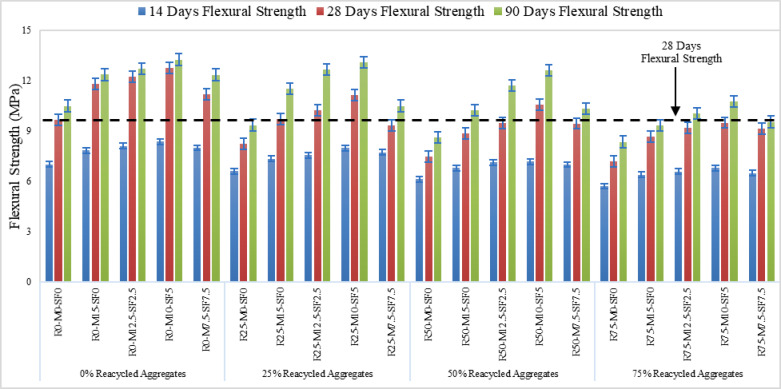
Flexural Strength of Concrete.

**Fig. 12:**
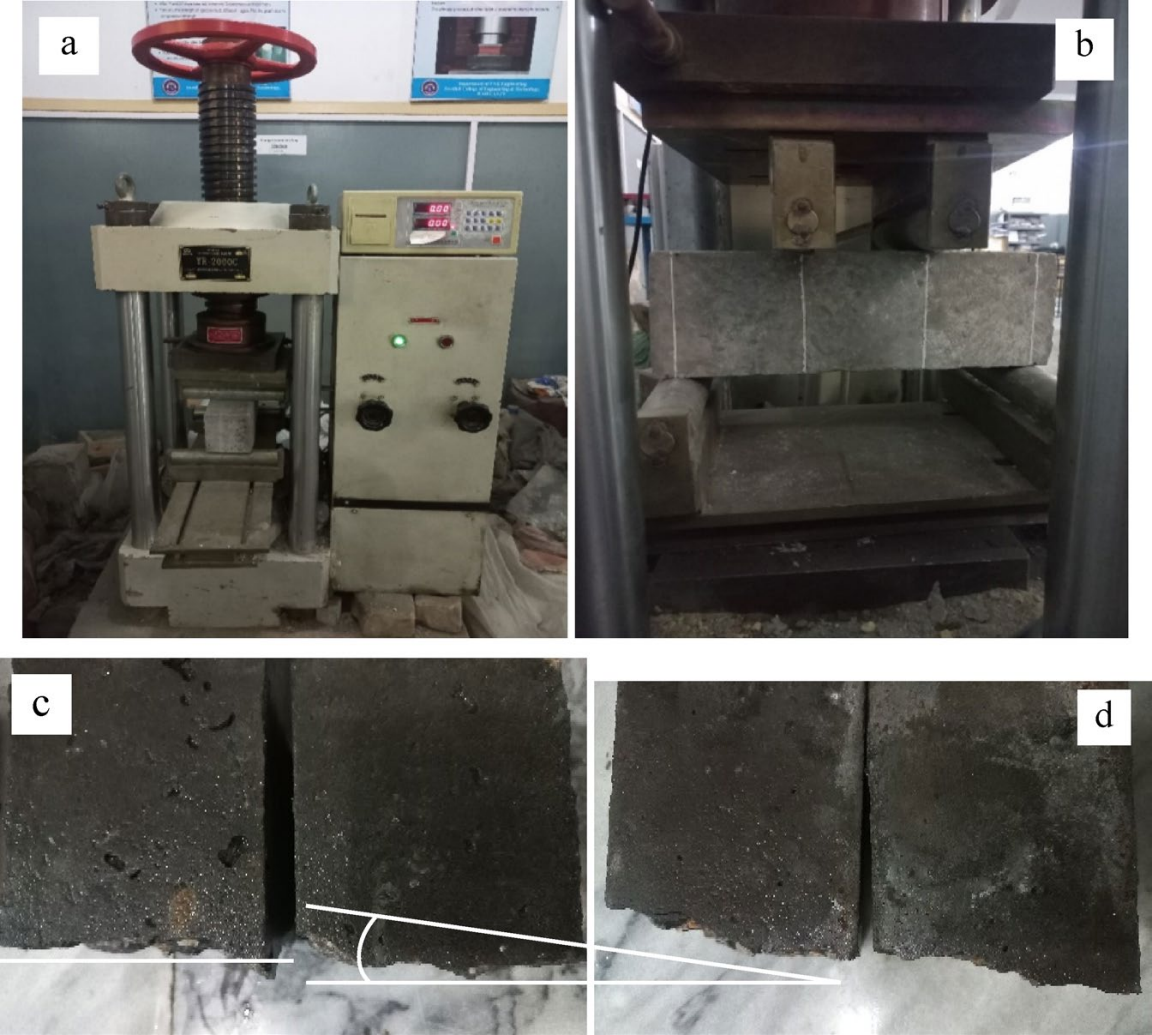
(**a**) Sample in Testing Machine; (**b**) Markings; (**c**) Linear Cracks in Natural Aggregates Concrete; (d) Diagonal Cracks in Recycled Aggregates Concrete.

Figures 9 and 11 represent the split tensile and flexural strength test results on selected concrete samples. It demonstrates that the variance in both strengths of several concrete mixes with varying percentages of recycled aggregate (0, 25, 50, and 75%) and curing times (14 days, 28 days, and 90 days). The strength of all concrete mixes improves dramatically as the curing period increases from 14 to 28 days and up to 90 days. This pattern is likely due to the fact that concrete increases strength with time because of increased cement hydration. Split Tensile strength and flexural strength decreases as the percentage used of recycled aggregates in the mix increases. Figure 10(a) and Fig. 12(a & b) shows the concrete specimen under testing machine while Fig. 10 (b & c) Shows crack pattern for split tensile test and Fig. 12 (c & d) shows crack pattern of flexural test. For all curing times, mixes with 0% recycled aggregates (R0) have the maximum strength, while mixtures with 75% recycled aggregates have the lowest. In concrete samples with R75-MK0-SF0 samples showed a 17.6% reduction and a 19.72% rise with R50-MK10-SF5 in split tensile strength when compared to control samples at 90 days of curing. Similarly, the strength reduction in flexural with R75-MK0-SF0 was noticed as 25.67% and the rise was 9.31% with R50-MK10-SF5 when compared with R0-MK0-SF0. The drop in strength is likely due to the high-water absorption of RA concrete and porosity of mortar on the RA surface. It is also inferred that tensile strength diminishes with increasing recycled aggregate content, probably because to the poorer link between recycled aggregates and cement matrix compared to natural aggregates.

Metakaolin and silica fume increase strength in all mixes up to the combination of 10% metakaolin and 5% silica-fume. This is due to the fact that after a certain percentage i.e. optimal percentage pozzolanic materials do not add to the hydration process and remain as a filler material which actually cause this reduction. Mixes with SCMs, such as R0-MK10-SF5 and R50-MK10-SF5, have much greater strengths than mixes without SCMs. Tensile strengths are highest in mixes containing moderate to high amounts of metakaolin and silica fume (MK10-SF5), demonstrating that SCMs increase the overall tensile performance of concrete.

#### Statistical analysis of split tensile and flexural Stength

**Fig. 13:**
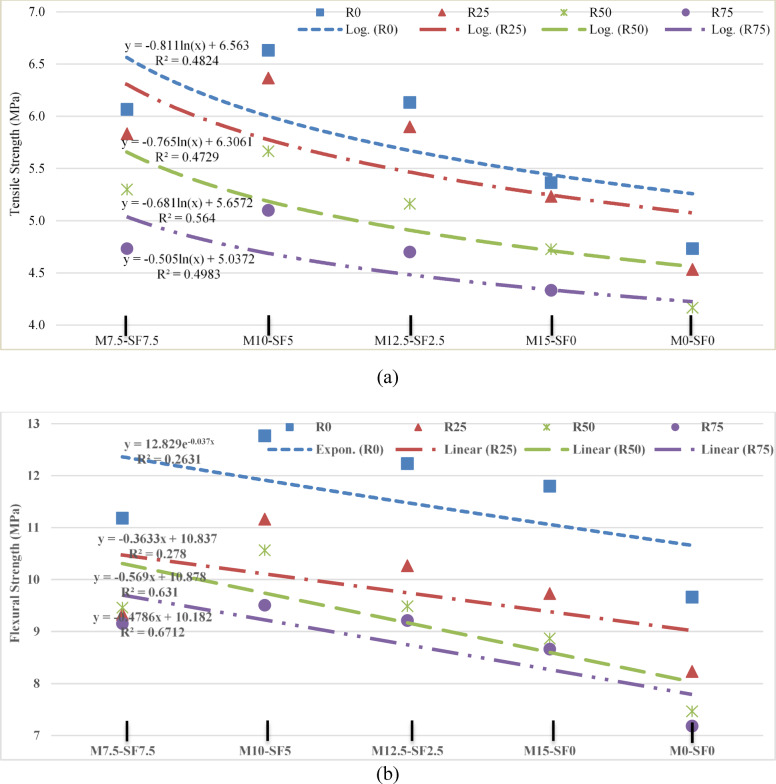
(**a**) Statistical Analysis of Tensile Strength of Concrete (**b**) Statistical Analysis of Flexural Strength of Concrete.

A logarithmic curve for tensile Fig. 13 (a) and linear fit was used for flexural strength Fig. 13 (b) was used to statistically confirm the outcomes. The line was seen closely packed to the locations in the correlation analysis. The study of correlation shows with great precision that concrete strength utilizing 0% of RA was the highest, despite the inclusion of MK or SF. It is also demonstrated by statistical analysis that MK and SF in recycled aggregate concrete greatly increased the strengths by mitigating the negative impacts of RA. The current study’s strength is consistent with earlier research findings.

### Water absorption

**Fig. 14:**
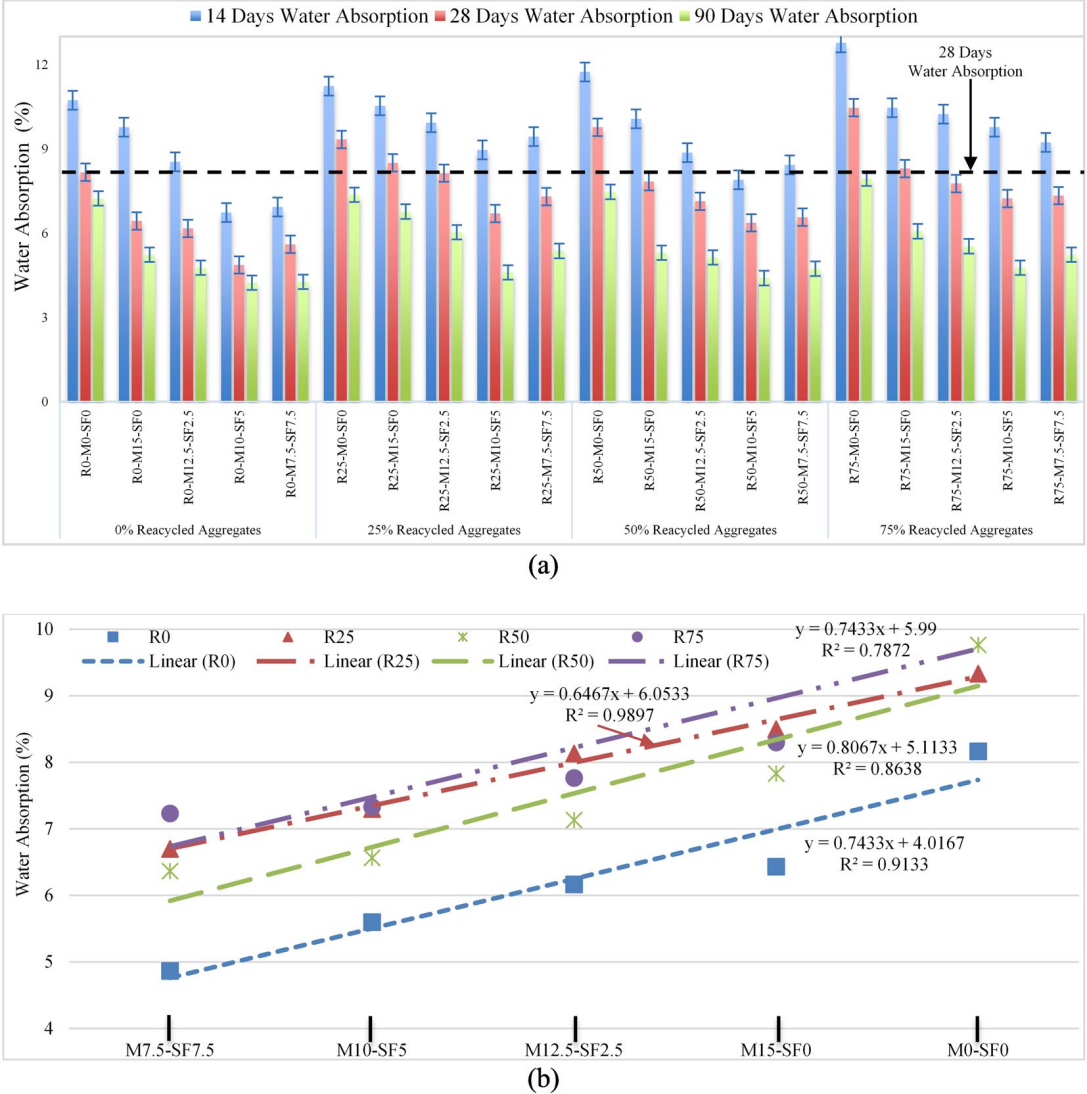
(**a**) Sample in Oven; (**b**) 24 h Heating; (**c**) Natural Aggregates Concrete Weight; (**d**) Recycled Aggregates Concrete Weight.

**Fig. 15:**
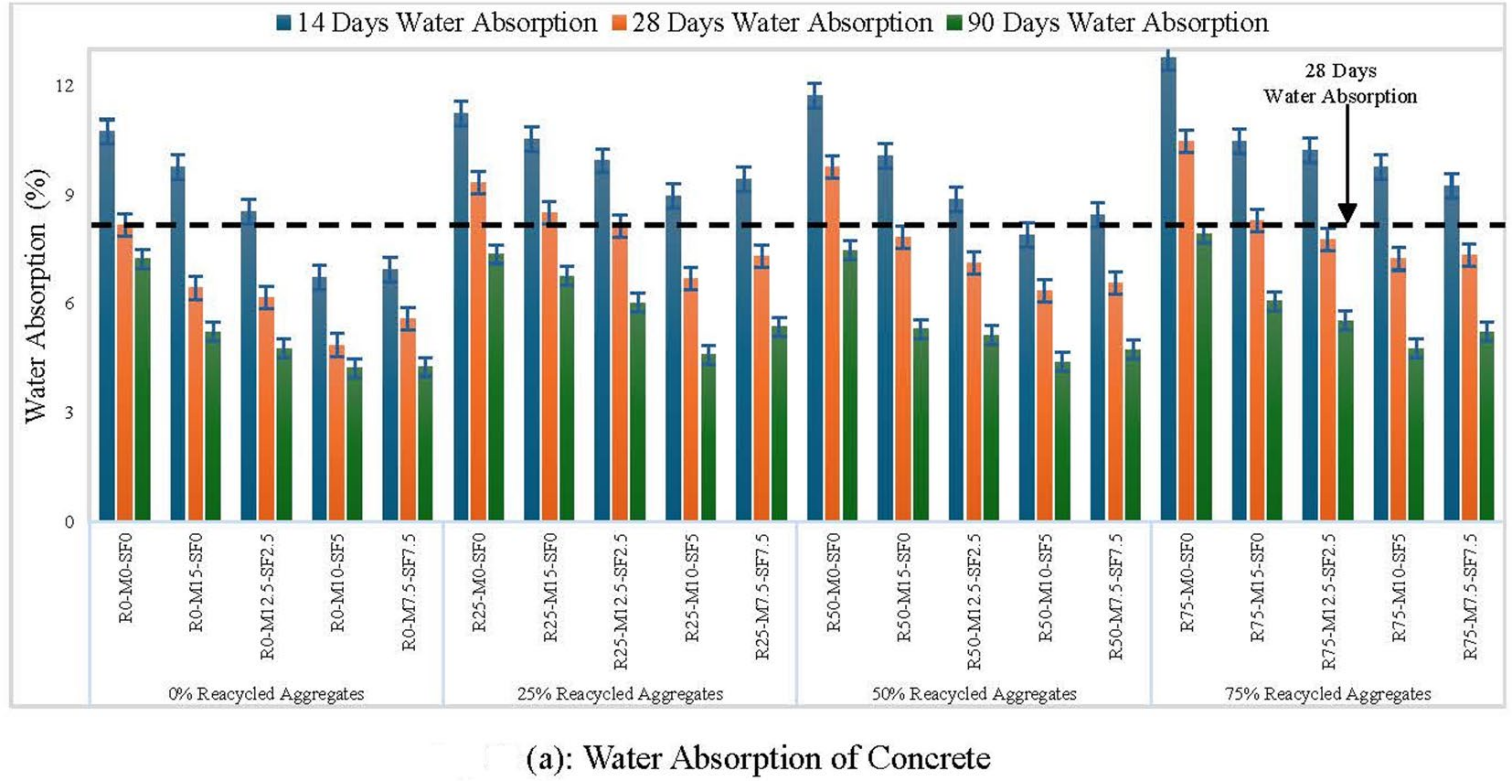
(**a**) Water Absorption of Concrete (**b**) Statistical Analysis of Water Absorption of Concrete.

Water absorption experiment can be performed to determine concrete pores analysis and its porosity. Voids abundance and their connectivity affect the overall volume of permeable pores in concrete. This experiment was carried out on samples by immersing them in water for up to 90 days and Fig. 14 (a-d) shows experimental setup to determine the water absorption. Water absorption results of several samples are depicted in Fig. 15 (a). for 28 days of curing, soon the RA concentration grew from 0 to 75%, water absorption increased to 28.27%. However, with SCMs, it decreases, with a maximum drop of 20.87%, represented with an R50-MK10-SF5 combination. Due to the porous and permeable old mortar found in RCA contributes to an increase in water absorption as RA increases. Using 10% metakaolin and 5% silica-fume decreased water absorption by 10.6%, 20.87%, and 10.2% for 25%, 50%, and 75% RA, respectively, when confronted to concrete with no recycled aggregates, metakaolin and silica-fume, as statistically illustrated in Fig. 15 (b).

### Acid resistance

**Fig. 16:**
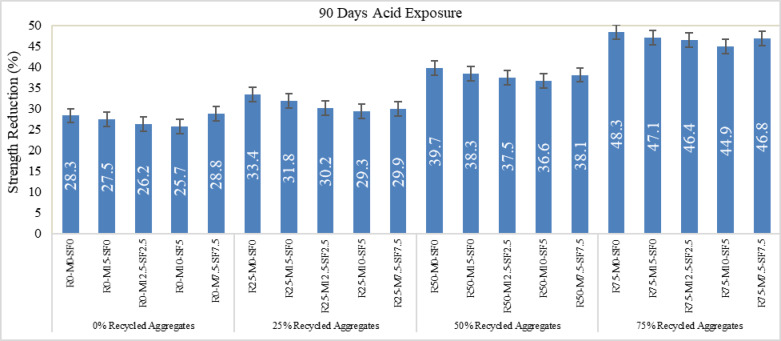
(**a** & **b**) H_2_SO_4_ Curing; (**c**) Apparent Deterioration.

**Fig. 17:**
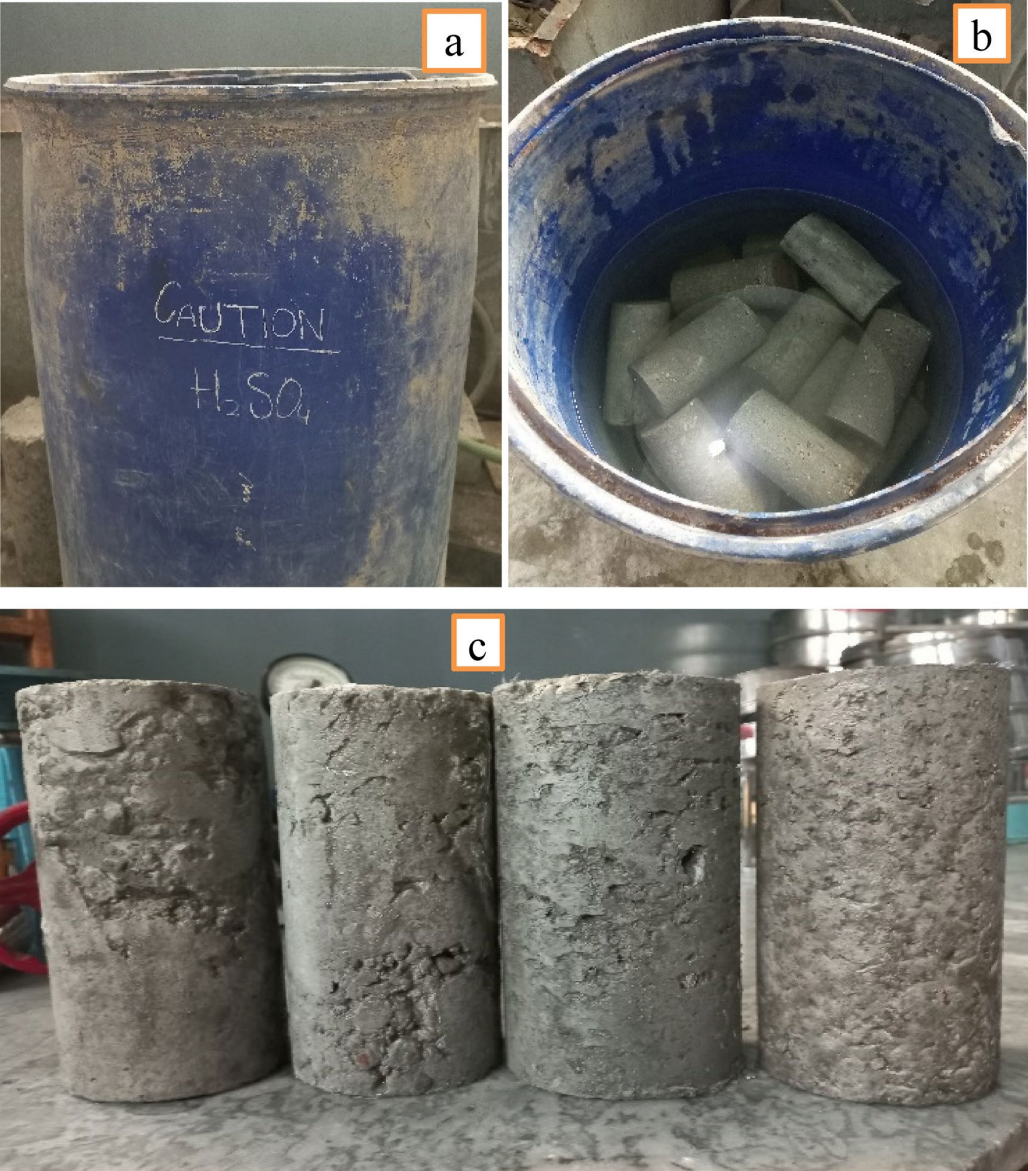
Strength reduction in percentage at 90 days Acid Exposure.

Acid resistance of RAC was tested on 14 days strength. All mixes were first cured in normal water for 14 days and then placed in sulfuric acid (5 mol solution) for the next 90 days as shown in Fig. 16 (a & b). The molarity of solution was being checked regularly to maintain the uniformity. After 90 days exposure to the acidic environment the samples were tested under a compression machine. The strength properties of RAC concrete containing MK at 10% and SF at 5% display better acid resistance for 5% sulfuric acid solution. Sulfuric acid markedly damages cement-based materials by interacting with calcium hydroxide (CH) to transform it into gypsum (CaSO₄0.2 H₂O) which triggers expansion and causes material cracking and surface degradation (Fig. 16c). Sulfate ions react within tricalcium aluminate (C₃A) cement to produce expansive ettringite which induces structural disintegration through internal stress. Within cements C-S-H gel acts as the principal binding agent yet breaks down over time when exposed to acids which causes material strength to decrease^52^. Adding MK and SF produces concrete with enhanced acid resistance capabilities beyond normal RAC design by creating less mass loss and leading to superior long-term durability. The addition of MK and SF enhances resistance but fails to achieve a state of absolute protection towards deterioration. Strength reduction in percentage of different mixes is shown in Fig. 17.

It can be seen from the result that acid resistance of RAC reduces between 25.7% and 48.3% as the recycled coarse aggregate content increases from zero to 75%. Concrete specimens experience the maximum deterioration among those without SF yet specimens containing 5% SF exhibit enhanced durability throughout every level of RCA replacement. The M10-SF5 group demonstrated reduced strength by 25.7% at 0% RCA while strength losses increased progressively to 29.3% at 25% RCA before reaching 36.6% at 50% RCA and reaching the most severe reduction at 46.4% when using 75% RCA. Strength measurements show a 3–10% strength decrement each time the RCA content increases by 25%. The durability of specimens falls most sharply during the transition from 50 to 75% RCA content. This confirms that higher RCA concentrations weaken concrete. Hence, metakaolin and silica fume provides protection against acid deterioration yet significant durability loss occurs at RCA contents above 50%, requiring thorough mix design modifications.

### Ultrasonic pulse velocity (UPV)

**Fig. 18:**
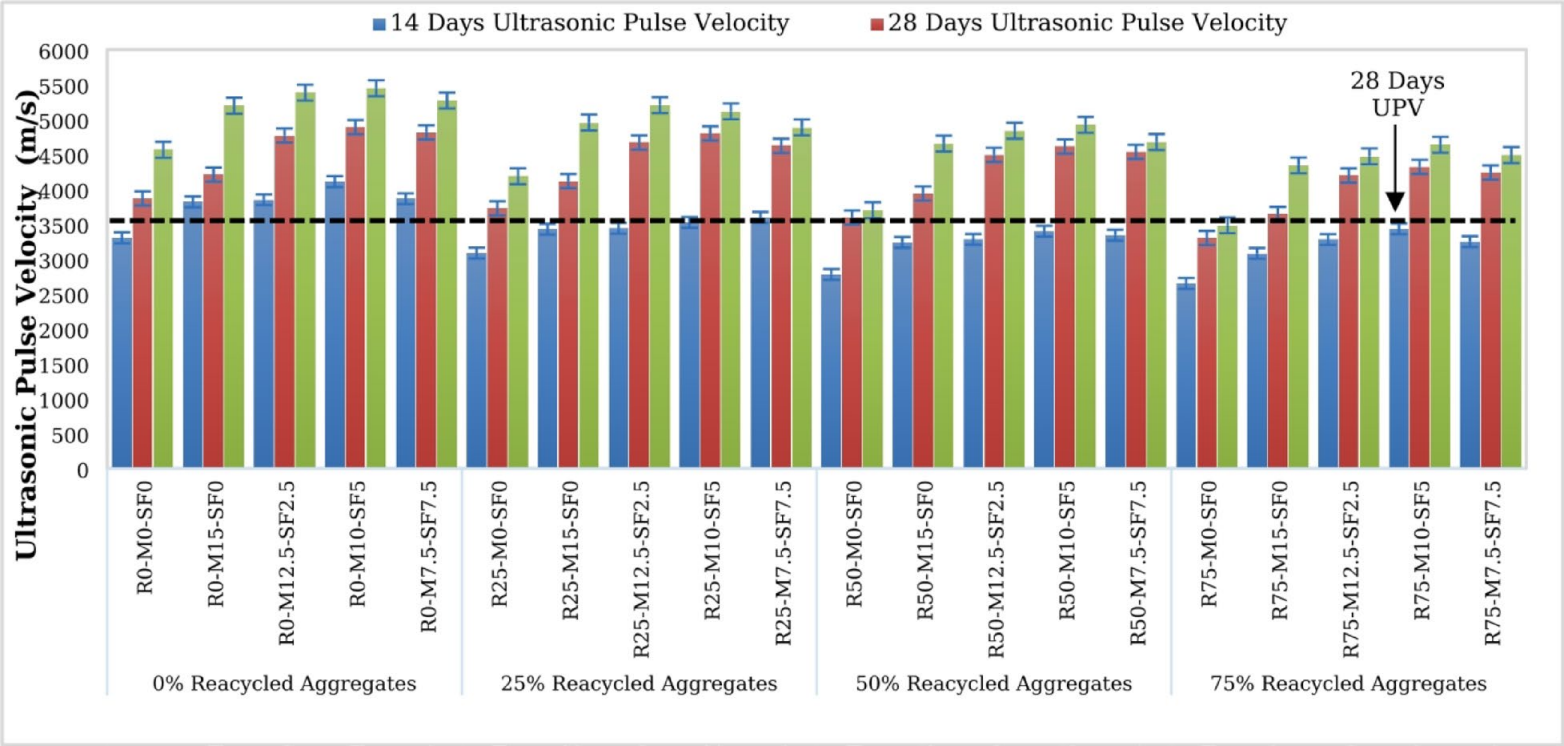
Ultrasonic Pulse Velocity of Concrete.

**Fig. 19:**
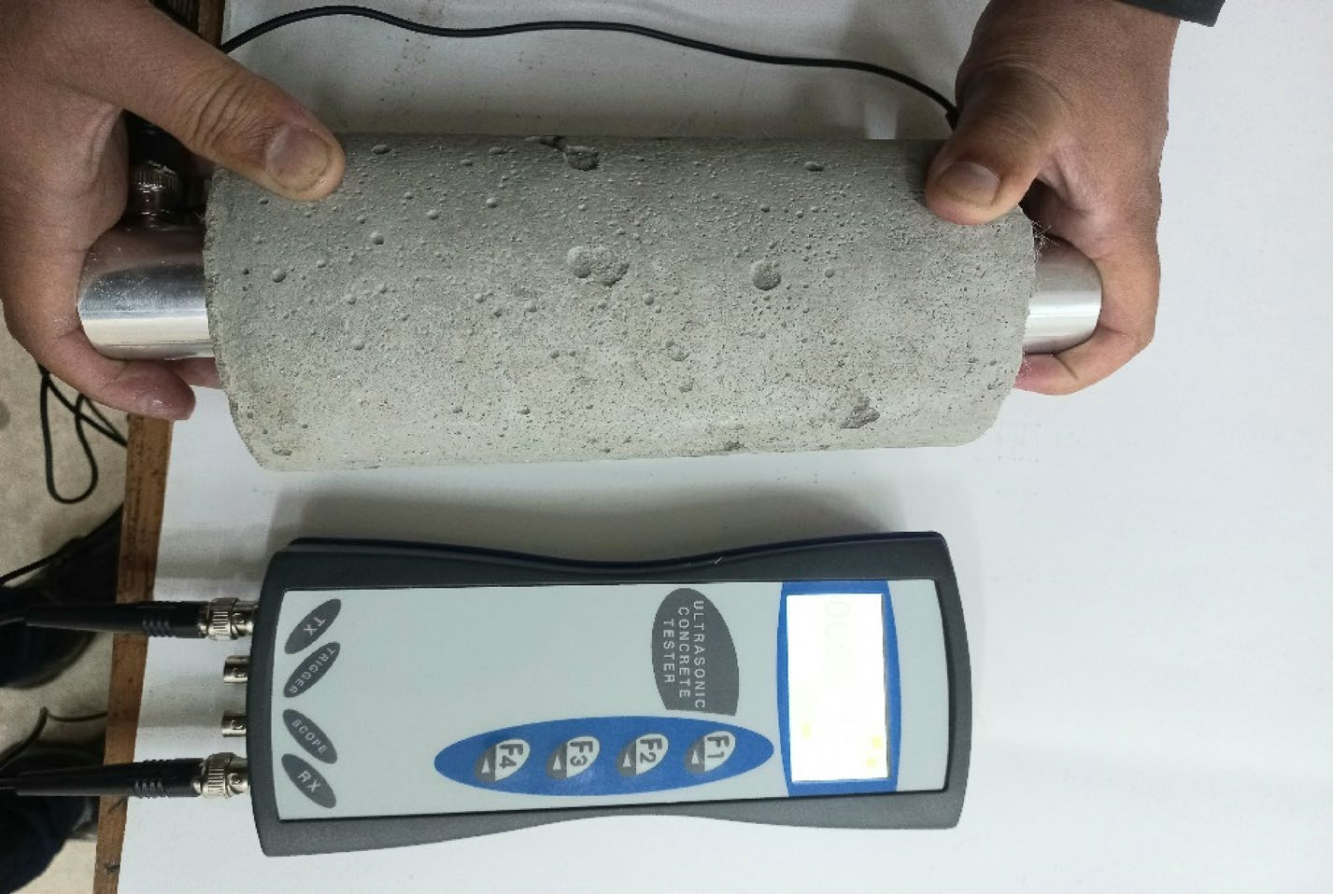
UPV test of specimen.

The ultrasonic pulse velocity (UPV) test on the concrete specimens at 7, 28, and 90 days was carried out having various percentages of RCA and MK-SF, as shown in Fig. 18. The mean UPV value of 90 days samples is 4565.7 m/sec for the control samples, but the various concentrations of the RCA revealed UPV values of 4184.8 m/sec, 3697.7 m/sec, and 3481.5 m/sec for 25% RA, 50% RA, and 75% RA, respectively. Overall, all mixes meet the ‘good’ condition requirement, with the RA0-MK10-SF5 mix meeting the ‘outstanding’ standard. Because of the degrading characteristics of concrete interior particles, pore structures in concrete mixes had a detrimental influence on UPV values and quality evaluation^53^. However, the concrete mixes with up to 50% RCA acceptable UPV values are classified as ‘good’ in the concrete quality evaluation. The UPV values of concrete samples ranging from 0% RA to 75% RA were determined to be 23.75% lower. The normalized UPV value study demonstrates that the R25-MK10-SF5, R50-MK10-SF5, and R75-MK10-SF5 mixes achieve 95.54%, 90.35%, and 85.11% RAC0-MK10-SF5 pulse velocity at 90 days, respectively. Furthermore, the comparable degrading trend of samples with different RCA concentrated mixes were discovered at 14 and 28 days of curing, as RCA fragments increased the quantity of trapped air in the concrete mix, impairing ultrasonic wave propagation^54^. The UPV intensities of the R25, R50, and R75 mixtures decrease as the RCA concentration increases. These ultrasonic pulse velocity strength ratings are shadows by aggregate density and the concrete’s interfacial transition zone (ITZ), hence weaker ITZ and more porosity result in lower values. The standard deviation of the UPV data is revealed by statistical analysis, which is 14.799, with coefficients of variation ranging from 0.003 to 0.015, and standard errors ranging from 0.302 to 1.274. The 95% confidence interval analysis yielded the lowest and greatest UPV values of 4023 and 4915 m/sec, respectively. This type of study demonstrates that with a larger percentage replacement, data variances rise due to the possibility of increased pore space buildup, poor bonding between the adhering mortar and aggregate, and an internal sample fracture in the specimen. The testing performs shown in Fig. 19.

### Fire performance

**Fig. 20:**
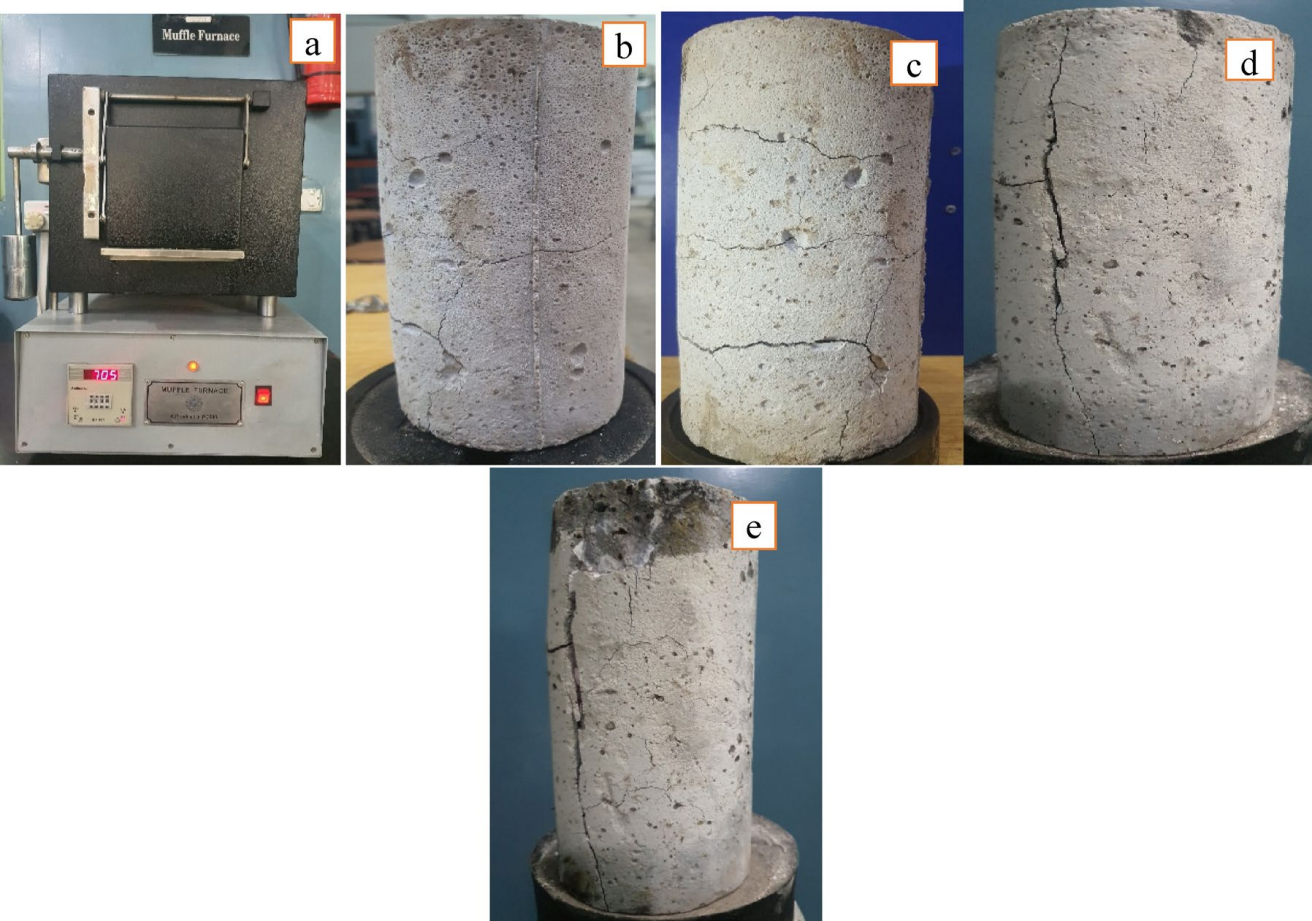
(**a**) Specimen in muffle Furnace; (**b**) Cracks at 300^o^C; (**c**) Cracks at 500^o^C; (**d**) Cracks at 700^o^C; (**e**) Cracks at 900^o^C.

**Fig. 21:**
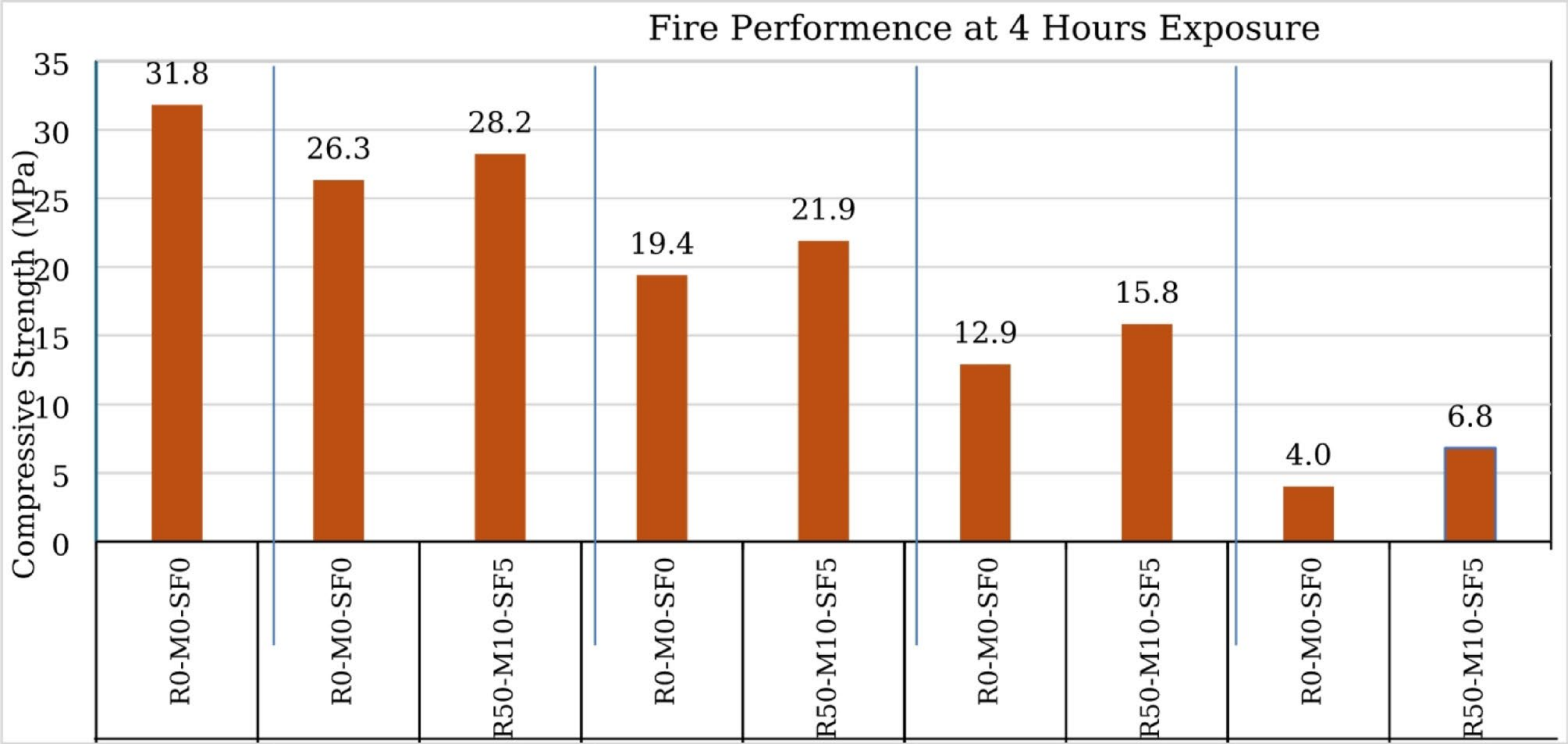
90 Days Fire Performance of Concrete at Different Temperatures.

Fire performance of RAC was tested using the muffle furnace, specimens were exposed to different temperatures (300^o^C, 500^o^C, 700^o^C and 900^o^C) for 4 h and then allowed to cool down naturally in the furnace. After that strength reduction in both NAC and RAC was calculated and cracks in concrete was analyzed. At each temperature three samples of both NAC and RAC (M50-M10-SF5) were placed in furnace (Fig. 20a). From the results (Fig. 21) it is calculated that 17% in NAC and 15.8% in RAC, 39.2% in NAC and 34.5% in RAC, 59.3% in NAC and 52.8% in RAC and 87.4% in NAC and 79.7% in RAC reduction occurred in concrete samples at 300^o^C, 500^o^C, 700^o^C and 900^o^C temperature respectively when they were exposed to fire for 4 h. The results show that with increasing the temperature, the strength reduction in NAC is more and RAC shows better performance than RAC (similar results are also illustrated by other researchers^55–57^). The strength reduction of concrete under the fire is due to the thermal, chemical, and physical changes in the structure of concrete. At increased temperatures, both free and bound water in the cement matrix evaporate, resulting in shrinkage and microcracking. Hydration products such as calcium silicate hydrate (C-S-H) and calcium hydroxide breakdown at temperatures ranging from 300 to 600 °C, causing flaws in the cement paste. Internal stresses are formed as a result of the thermal expansion mismatch between aggregates and cement paste, which causes further cracking Fig. 20 (b & c). Damage is exacerbated by phase shifts in aggregates: quartz swells about 573 °C and carbonate decomposes at 700–900 °C.

Steam builds up inside the concrete, causing pieces to fall off Fig. 20(d & e), thereby lowering the cross section. Above 800 °C, the microstructure becomes porous and brittle, with full loss of load bearing capacity Fig. 20 (e). This deterioration is caused by a mixture of several factors, which reduce the concrete’s integrity. However, the better performance of RAC with metakaolin and silica-fume is likely due to the fact that metakaolin and silica fume increase concrete’s thermal resistance, both through better microstructure and resistance to heat-induced deterioration. Also this might be due to the fact that metakaolin, a highly reactive pozzolanic material, combines with calcium hydroxide (Ca (OH)₂) in the cement matrix to produce calcium silicate hydrate (C-S-H), which enhances density and lowers porosity^58^. Another probability is that the finer microstructure restricts the routes via which heat and steam may pass through concrete, reducing the likelihood of microcracking caused by fire exposure. By filling in pores and micro-voids, silica fume and other fillers create a densely packed structure that is resistant to heat damage, thereby densifying the matrix. Also strengthening the link between the aggregate and cement paste, metakaolin and silica fume work together to lessen the thermal stresses caused by differential expansion. By reducing the likelihood of spalling and delaying the breakdown of important cement hydration products, this combination improves strength retention at elevated temperatures.

## Conclusions and recommendations

This research is based on observing the simultaneous effects of recycled aggregates, metakaolin and silica-fume on the mechanical properties of concrete following the fire performance and the following conclusions are extracted:

1. Using recycled aggregate (RA) instead of natural aggregate (NA) impacts concrete’s mechanical properties and water absorption at any curing age. However, Metakaolin and silica fume mitigate these impacts by stiffening the concrete matrix and improving hydration processes.
2. Optimum dosage of MK and SF in such combination is observed 10% and 5% respectively with 50% RCA content as all concrete parameters shows the positive indications.
3. 28 days compressive strength, tensile strength and flexural strength was 5.19%, 16.47% and 8.52% higher respectively when utilizing 10% MK, 5% SF and 50% of Recycled aggregate over controlled sample.
4. The ultrasonic pulse velocity of R50-MK10-SF5 concrete at 28 days is 16.13% higher than R0-MK0-SF0 concrete emphasis the solidification of its structure and strong bond between its constituents. In addition to 20.87% less water absorption than the NAC confirming the densification and enhanced durability of concrete.
5. RCA content influences acid resistance negatively when reaching 75% RCA shows maximum deterioration. Metakaolin and silica fume strengthen concrete structure but exceeding a 50% RCA ratio leads to noticeable strength reductions that need special considerations.
6. The fire resistance of R50-MK10-SF5 concrete was also slightly higher than virgin concrete at all temperature levels showing that the metakaolin and silica-fume in this combination not only improve other parameters of concrete but also enhance the fire performance.

Therefore, it can be summarized from the above conclusions that metakaolin and silica-fume with the combination of MK10-SF5 is the valuable substitute of cement as well as excellent performance booster when recycled aggregates up to 50% are incorporated into the concrete. These properties are improved most likely due to the additional hydration utilizing the SCMs thus producing strong bond between concrete constituents, improvements and densification of concrete matrix as elaborated by other researchers in similar studies. Keeping in mind all the positive outcomes of this study, such concrete can be studied further at microstructure level (XRD, FTIR and SEM etc.) as it has shown sparkling feature and can be used in structural application in future.

## Data Availability

The data is available from the corresponding author upon request.
